# Understanding patient perspectives on digital therapeutics and its platform for insomnia: insights from focused group interviews

**DOI:** 10.1186/s12913-024-11286-4

**Published:** 2024-07-29

**Authors:** Jinhyun Kim, Kyung Mee Park, Suonaa Lee, Sooji Park, Minji Hong, Jaeyong Shin, Eun Lee

**Affiliations:** 1 Department of Preventive Medicine, Yonsei University College of Medicine, 50 Yonsei-ro, Seodaemun-gu, Seoul, 03722 Republic of Korea; 2 Institute of Health Services Research, Yonsei University, Seoul, Republic of Korea; 3 Department of Hospital Medicine, Yongin Severance Hospital, Yonsei University College of Medicine, Yongin, Republic of Korea; 4 Department of Psychiatry and the Institute of Behavioral Science in Medicine, Yonsei University College of Medicine, 50-1 Yonsei-to, Seodaemun-gu, Seoul, 03722 Republic of Korea; 5 Institute for Innovation in Digital Healthcare, Yonsei University, Seoul, Republic of Korea; 6 Department of Medical Device Engineering and Management, Yonsei University College of Medicine, Seoul, Republic of Korea

**Keywords:** Digital technology, Insomnia, Qualitative research, Focus group, Patient preference

## Abstract

**Background:**

Digital therapeutics (DTx) is a treatment option that uses computer software to provide evidence-based interventions for medical disorders. DTx platforms are digital services that facilitate interactions among stakeholders of DTx treatment within a standardized structure. However, there is still a lack of overall awareness regarding the effectiveness and usage of DTx and DTx platforms. This study aimed to investigate insomnia patients’ recognition, thoughts, feelings, and demands for conventional treatments versus DTx for insomnia.

**Methods:**

Nine participants, aged 19–50 years, who had experience with professional medical interventions for insomnia, were recruited through purposive sampling. Two online focus group interviews, each lasting 1.5 h, were conducted. The interview questions focused on difficulties encountered during conventional treatment, inadequate recognition of DTx, and concerns and demands regarding DTx and its platform. The data were analyzed using thematic analysis.

**Results:**

The participants reported subjective difficulties associated with receiving conventional treatment, including concerns about drug side effects and dependence, social stigma, and lack of perceived necessity for treatment. They expressed concerns about DTx, such as cost-effectiveness, evidence on efficacy, and concerns about breach of personal information. Additionally, their demands included convenience of use, reduction in social stigma related to the use of DTx, compatibility of DTx with other healthcare systems, and enhanced communication with healthcare providers when using DTx platforms.

**Conclusions:**

The focus group highlighted the need for increased awareness, demonstrated efficacy, cost-effectiveness, cybersecurity measures, and accessibility of insomnia DTx and its platforms. Tailored approaches considering patient characteristics are crucial for widespread adoption of insomnia DTx and its platforms.

**Supplementary Information:**

The online version contains supplementary material available at 10.1186/s12913-024-11286-4.

## Background

Digital therapeutics (DTx) are an emerging treatment option characterized by personalization, offering an alternative to pharmacological therapies [1]. According to the Digital Therapeutics Alliance, an international organization defining and setting standards for DTx, DTx is a type of “Software as a Medical Device” (SaMD) that provides evidence-based therapeutic interventions for medical disorders [1]. According to the European Data Protection Supervisor, DTx, as a type of SaMD, has a proven clinical benefit [2]. DTx is distinguished from conventional pharmacotherapy by its relatively low development cost, short treatment period, minor side effects, high management adherence, and real-time automatic monitoring [3]. Moreover, DTx is different from wellness applications (digital health), which are software programs for improving general health, not treating or managing diseases, and clinical evidence is not required for wellness applications [4]. As of April 2023, several DTx products, supported by real-world evidence, satisfy the core principles based on the data of the Digital Therapeutics Alliance, with clinical evidence reviewed by regulatory bodies (Table 1) [1].

**Table 1:** List of digital therapeutics that satisfy core principles of Digital Therapeutics Alliance

Product name	Company	Target disease(s)	Outcomes	Approval
BlueStar	Welldoc	Type 1 and 2 diabetes	Decrease in Glycated hemoglobin (HbA1C)	USA, Canada
CureApp HT	CureApp	Essential Hypertension	Decrease in Systolic blood pressure	Japan
d-Nav	Hygieia	Type 2 diabetes	Decrease in HbA1C	USA, EU
Dario Blood Glucose Monitoring System	Dario Health	Type 1 and 2 diabetes	Decrease in HbA1C	USA, Europe, Canada, Australia, Israel
Daylight	Big Health	Generalized anxiety disorder	Anxiety improvement	USA, UK
deprexis	Orexo	Depression	Depressive symptoms improvement	USA, Germany, Switzerland
EndeavorRx	Akili	Attention-deficit/hyperactivity disorder (ADHD)	Sustained and selective attention improvement	USA
Freespira	Freespira, Inc.	Post-traumatic stress disorder (PTSD), panic disorder	Decrease in panic attacks and PTSD symptoms	USA
HelloBetter Chronic Pain	GET.ON Institut für	Chronic Pain	Decrease in pain interference, quality of life improvement	Germany, Switzerland
HelloBetter Diabetes and Depression	GET.ON Institut für	Depression among diabetes patients	Decrease in depressive symptoms	Germany, Switzerland
HelloBetter Panic	GET.ON Institut für	Panic disorder, Agoraphobia	Decrease in anxiety symptoms and agoraphobic cognitions	Germany, Switzerland
HelloBetter Stress and Burnout	GET.ON Institut für	Stress. burnout	Decrease in emotional distress	Germany, Switzerland
HelloBetter Vaginismus Plus	GET.ON Institut für	Organic Vaginismus, Non-Organic Vaginismus	Decrease in penetration-related anxiety, genital pain, pain related sexual and non-sexual impairment	Germany, Switzerland
Insulia	Voluntis	Type 2 diabetes	Blood sugar control through Insulin dosage recommendation	USA, EU, Canada
Kaiku Health	Kaiku Health	Cancer	Earlier detection of symptoms through non-urgent communication with medical professional	EU, USA, Australia, New Zealand, Canada, South Africa, Argentina, Mexico, Japan
Leva	Axena Health	Urinary and fecal incontinence among women	Decrease in incontinence symptoms	USA
Nerivio	Theranica	Migraine	Pain relief	EU, USA
Propeller	Propeller Health	Chronic obstructive pulmonary disease (COPD), Asthma	increase in medication adherence, decrease in rescue inhaler use	USA, Canada, EU
reSET	Pear Therapeutics	Substance use disorder	Increase in abstinence rates, rates of retention	USA
reSET-O	Pear Therapeutics	Opioid use disorder	Increase in retention rates (as adjunct management to buprenorphine treatment)	USA
SC Nicotine Addiction Treatment App and CO Checker	CureApp	Nicotine use disorder	Increase in smoking cessation rate	Japan
Sleepio	Big Health	Insomnia	Insomnia improvement	USA, UK
Somryst	Pear Therapeutics	Chronic insomnia	Sleep initiation and maintenance improvement	USA
SparkRx	Limbix	Depression in adolescents	Decrease in depressive symptoms in adolescent	USA
TALi	Tali Digital	Attention impairment	Attention and communication skill improvement	Australia, EU, USA
vorvida	Orexo	Alcohol use	Decrease in daily alcohol consumption	USA

According to the Ministry of Food and Drug Safety (MFDS) of South Korea, DTx is defined as an SaMD that provides evidence-based interventions targeting medical disorders and/or diseases. DTx was first approved in South Korea as an application for insomnia symptoms [5, 6]. In addition, several DTx are under exploratory or confirmatory studies, including DTx for respiratory rehabilitation, insomnia, alcohol use disorder, and suicide among patients with depression [7]. Meanwhile, based on a survey of several medical institutes and DTx companies in 2022, a high demand exists for connectivity between DTx and medical institutes through electronic medical records and data sharing of medical test results or patient compliance with DTx [8, 9]. Therefore, an online digital service platform is needed to facilitate interaction, compatibility, and communication within a standardized medical structure among patients, medical institutes, DTx providers, and relevant government authorities. For example, a DTx platform could generate standardized usage and clinical data pertaining to various DTx interventions targeting the same disease(s) or symptom(s) and seamlessly transfer this data to the electronic medical record systems of medical institutions with prior patient consent. Furthermore, the platform could have the capability to transfer patient medical data from independent medical institutions to DTx software and the Korea National Health Insurance system (NHIS) with prior patient consent, thus facilitating the personalization of DTx and preventing overlapping of prescriptions.

Insomnia is a highly prevalent condition in South Korea, with over 30% of the population reporting insomnia symptoms and approximately 6% seeking clinical treatment for sleep disorders [10, 11]. Conventional therapies for insomnia encompass pharmacological options such as benzodiazepines, zolpidem, and antidepressants, as well as non-pharmacological interventions such as in-person cognitive behavioral therapy for insomnia (CBT-I), incorporating techniques like sleep restriction, stimulus control, and patient education [12]. Although proven effective, these traditional management strategies face inherent limitations, including potential adverse effects and dependence risks associated with medications and restricted access to in-person CBT-I due to a scarcity of trained providers [12].

Insomnia DTx without in-person supervision has been used as an effective alternative for insomnia management [13, 14]. In 2017, a randomized clinical trial demonstrated the efficacy of insomnia DTx in 303 adults, with 56.6% insomnia remission [15]. Additionally, another randomized clinical trial indicated that DTx for insomnia can enhance psychological well-being, sleep quality, and alleviate insomnia symptom [14]. However, despite growing public interest, the overall awareness regarding the effectiveness and usage of DTx and its platforms remains limited. A 2021 survey of 933 Koreans with diseases found 50.8% roughly understood digital healthcare systems, 76.8% agreed they were necessary, but only 18.9% had experienced DTx. The main concerns were medical errors (51.8%) and data privacy breach (19.4%) [9]. Due to the relatively low recognition of DTx, the opinions of potential users regarding the concerns and demands for conventional treatments and DTx may not be accurately reflected in the current literature on the advancement of DTx and its platform.

Given that insomnia DTx is a recently developed and rapidly advancing alternative treatment in Korea, it is essential to consider patients’ subjective evaluations for its successful integration. However, the opinions of insomnia patients have been relatively overlooked compared to other stakeholders, such as physicians and developers. Therefore, understanding patients’ concerns and the reasons for the low recognition of DTx is crucial for its successful adoption as an alternative treatment. The aim is to understand patients’ recognition, thoughts, feelings, and demands regarding DTx and its platform. Our primary focus was on insomnia DTx, which has seen significant advancements in the field of DTx. Additionally, it addresses issues associated with traditional psychiatric management, such as social stigma [16, 17]. To gather comprehensive insights, we conducted focus group interviews (FGIs) with participants who had experience with professional medical interventions for insomnia.

## Methods

### Study participants

Purposive sampling was utilized to ensure optimal interaction and group dynamics among participants and interviewers and to recruit information-risk participants [18]. There were no prior relationships between the participants themselves or between the participants and the researchers. The selection criteria were based on shared health-related and socio-demographic characteristics [19]. The study inclusion criteria were as follows: aged 19–50 years, experience with professional medical intervention for insomnia symptoms through medical institutes, and familiar with the use of the online meeting programs. The eligibility criteria for insomnia were determined by asking participants, “Have you ever visited medical treatment for insomnia symptoms?” A total of 10 participants agreed to participate and were subsequently divided into two subgroups of five participants each, according to their age (around the age of 30 and around the age of 40), area of residence (Seoul or other areas), and occupation status. Prior to the interviews, JH sent an introductory email regarding the aim of the study, an overview of the funder and goals of the program (Ministry of Trade, Industry & Energy), and the interviewer’s occupation to 10 participants. One participant was excluded from the study as they did not respond to the introductory email and did not attend the scheduled interview for unknown reasons.

This study was approved by the Yonsei university health system, Severance hospital, Institutional review board (4-2022-1307). The reporting follows the guidelines outlined in the Consolidated Criteria for Qualitative Research (COREQ, Supplementary Tables 1 and 2).

### Interview and data collection

Between February and April 2023, two online FGIs were conducted using an online meeting platform (Microsoft Teams). The interviews were conducted remotely, with only the interviewers and approved participants present; participants joined from their home or workplace locations. The first FGI consisted of five participants, while the second FGI had four participants due to one participant not attending. The number of focus groups and the group size of five participants per focus group were determined according to previous research recommendations and to facilitate sharing of participants’ private medical symptoms among unfamiliar persons [20, 21]. Prior to the main discussion, the interviewer briefed the participants about DTx and its platforms (approved DTx platforms in the global market and South Korea as well as the anticipated functions of DTx platforms for patients, physicians, DTx manufacturers, and the NHIS), notified them about the discussion being recorded, and stressed on the significance of maintaining confidentiality. Each FGI lasted 1.5 h, with 4 or 5 participants sharing the interview duration by sequentially answering the interview questions. On average, each participant spent 15 min responding. The interview questions were determined according to the guidelines [22, 23]: open-ended questions, ordered in a natural flow, avoiding direct and quantitative questions, questions composed with “opening-introduction-transition-key-ending questions.” The team responsible for reviewing and interpreting interview data consisted of four distinguished experts: two qualified physicians specializing in board-certified psychiatry, a professional with DTx regulations from the MFDS in South Korea, and a preventive medicine specialist with expertise in DTx. JH (MD, psychiatrist and junior researcher, male) and SL (MD, psychiatrist and junior researcher, female) conducted the interview. The FGI questions were developed for this interview, and the questions are listed in Supplementary Table 3. The FGIs were not repeated and the FGIs were stopped after data saturation was achieved. Field notes were taken, and audio/video recordings were made during the interviews. The transcripts were not returned to participants for correction or additional comments after the interviews.

### Statistical analysis and data analysis

The mean and standard deviation of the participants’ ages were calculated using SAS software (Cary, NC: SAS Institute; Version 9.4). The interview data were subjected to thematic analysis [24, 25]. The two main researchers became familiar with the interview contents by repeatedly listening to the interview recording and reading the transcripts, including the verbally recorded nonverbal cues. Subsequently, they constructed an initial thematic framework based on the interview questions. The participants’ speeches were indexed and sorted based on the initial thematic framework. During the data extraction step, the framework was revised according to the final subthemes, which included indexed content. Thereafter, the themes were divided into subthemes. The data were summarized and displayed, and inadequate or unnecessary indices were deleted. The final data were subjected to abstraction and interpretation. All research team members reviewed the themes and subthemes. The interview participants were not asked to provide feedback on the findings.

## Results

Table 2 shows the general characteristics of the participants. The mean (standard deviation) age of the nine participants was 34.9 years (7.0 years). Seven participants reported having an occupation, while two participants reported being unemployed. Eight participants resided in urban areas (89%: six participants in Seoul, the capital of the Republic of Korea, and two participants in Gyeonggi-do), while one participant resided in a rural area (11%, Cheungcheongbuk-do). Three participants (33%) had never heard of DTx before, while six participants (67%) had heard of DTx but did not know the details. The mean duration of insomnia symptoms was 3.4 years (standard deviation, 3.7 years). Table 3 presents the themes and subthemes of the analysis.

**Table 2:** General characteristics of the study participants

Participant	Group	Age	Gender	Occupation status	Area of residence	Insomnia*	Duration	Recognition of DTx†
A	1	46	F	Employed	Seoul (urban)	Having insomnia	1 year	Low
B	1	38	F	Employed	Seoul (urban)	Having insomnia	7 years	Middle
C	1	46	F	Employed	Seoul (urban)	Having insomnia	3 years	Low
D	1	30	M	Unemployed	Seoul (urban)	Having insomnia	1 year	Middle
E	1	31	M	Employed	Seoul (urban)	Having insomnia	1 year	Low
F	2	27	M	Unemployed	Seoul (urban)	Having insomnia	1 year	Middle
G	2	31	M	Employed	Gyeonggi-do (urban)	Having insomnia	2 years	Middle
H	2	34	M	Employed	Gyeonggi-do (urban)	Having insomnia	3 years	Middle
I	2	31	F	Employed	Cheungcheongbuk-do (rural)	Having insomnia	12 years	Middle

*Abbreviation* DTx, Digital Therapeutics; N.A, not applicable

* All participants were insomnia patient with experience of professional psychiatric management

† Level of recognition of digital therapeutics before: Low, Never heard of DTx; Middle, Heard of DTx but does not know in detail; High, Familiar with DTx or had experience of DTx

**Table 3:** Themes and subthemes

Theme	Subtheme
Experienced subjective difficulty during conventional insomnia treatment	Concerns regarding drug side effects and dependence
	Social stigma
	Lack of medical management for insomnia
The reasons of inadequate recognition status for insomnia DTx	Scarcity of advertising of DTx
	Lack of medical management for insomnia
	Presence of conventional medical interventions
Concerns regarding DTx and DTx platforms	Concerns regarding DTx: Cost-effectiveness
	Concerns regarding DTx: Treatment efficacy
	Concerns regarding platform: Leakage of personal information
Demands regarding DTx and DTx platforms	Demands regarding DTx: Convenience and decrease in psychological burden
	Demands regarding platform: Compatibility and communication

*Abbreviation* DTx, Digital Therapeutics

### Theme 1: Subjective difficulties experienced during conventional insomnia treatment

#### Subtheme 1–1: Concerns regarding drug side effects and dependence

Several participants expressed significant concerns regarding the side effects of and dependence on medication for insomnia. For instance, they experienced somnolence during the daytime, dullness, or night walking. In addition, drug tolerance and dependence were associated with anxiety.

***Participant D*** I went to a psychiatry clinic first to get diagnosed and treated for my insomnia. I started taking Stilnox, and during the day, I feel extremely drowsy and like my mind is foggy. I feel like I’m becoming a little stupid. I also received sleep hygiene education, but it was challenging to follow. I was told not to look at my phone at night and to only sleep in bed, but I found it difficult to follow these guidelines.

***Participant I*** I work in broadcasting. I’ve found that taking medication can negatively affect my voice. One night, when I couldn’t sleep due to anxiety, I decided to try taking Stilnox, but the side effects were severe. Although I managed to fall asleep, I didn’t feel like I had a restful night’s sleep. I also experienced drug resistance and problems with sleepwalking at night. As a result, I stopped taking the medication altogether.

#### Subtheme 1–2: Social stigma

Most patients with insomnia faced psychological distress owing to social stigma and government regulations. The patients expressed concerns that seeking treatment at a psychiatric clinic or taking psychotropic medication would be perceived as indicators of mental illness. Additionally, they face obstacles due to policies governing psychotropic medication, including restrictions on the prescription duration of zolpidem.

***Participant A*** Because of the negative social perception and stigma associated with visiting a psychiatric clinic, some people may not even consider using DTx as an alternative treatment for insomnia. They may worry that seeking help for their sleep problems will make them seem as someone with a psychiatric disorder.

***Participant C***. *Even when I visit a mental health clinic*,* it’s inconvenient that medications like Stilnox can’t be prescribed in large amounts at once. I prefer to have the medication on hand and take it as needed*,* but I feel like there are limitations and concerns that if I am given a lot of medication*,* I might have negative thoughts about taking it excessively. As a result*,* I feel quite uncomfortable about it.*

***Participant F*** In Korea, to receive treatment for insomnia, one must visit a mental health clinic. However, the reality is that there is still social stigma and reluctance surrounding mental health treatment.

#### Subtheme 1–3: Lack of medical management for insomnia

Several participants held misconceptions regarding insomnia, perceiving it as a habitual problem or a natural occurrence common among humans rather than recognizing it as a medical symptom that necessitates professional management.

***Participant B*** I initially thought that I didn’t need to seek treatment for my sleeplessness and that trying meditation or other alternative methods found on YouTube might help. However, after receiving advice from people around me, I decided to seek treatment at a mental health clinic.

***Participant G*** I usually have difficulty falling asleep. I tend to take naps frequently during the day, but this makes it difficult for me to fall asleep at night. I am aware that my daytime napping is the root of the problem, but I find it challenging to adjust my lifestyle habits. I was unsure whether this was something that could be treated, and I lacked knowledge on the matter.

### Theme 2: Reasons for inadequate recognition status of insomnia DTx

#### Subtheme 2 − 1: scarcity of advertising for DTx

A few participants reported that they had never seen anyone around them using DTx nor had they heard any news about it. Furthermore, they did not have any knowledge of how DTx manages insomnia.

***Participant H*** It seems that there is not enough promotion about DTx. In fact, I knew that DTx existed, but I am not exactly sure what it does. Also, since I don’t know anyone around me who has experienced it, I don’t even consider it a treatment option.

***Participant I*** The name and definition of DTx are not clear. When I hear about it, I feel like something like a chip is going to be implanted in my brain. Older people in particular may not know much about it and may also have resistance to digital technology.

#### Subtheme 2–2: Lack of medical management for insomnia

A few participants emphasized that it is crucial for patients with insomnia to acknowledge the severity of their condition and actively seek treatment. Furthermore, they identified negative beliefs or schemas about insomnia as significant barriers to treatment in psychiatric clinics.

***Participant A*** Because of the negative social perception and stigma associated with visiting a psychiatric clinic, some people may not even consider using DTx as an alternative treatment for insomnia. They may worry that seeking help for their sleep problems will make them seem as someone with a psychiatric disorder.

***Participant F*** It seems that people don’t perceive insomnia as a serious illness. They don’t actively seek treatment for it. Patients need to be aware of the seriousness of insomnia as an illness and take it seriously.

#### Subtheme 2–3: Presence of conventional medical interventions

Well-established and evidence-based treatments for insomnia, including oral medication and cognitive behavioral therapy (CBT), are widely accepted. Consequently, patients who successfully improve their insomnia symptoms through these conventional interventions may be less likely to explore alternative options, including DTx.

***Participant E*** I already took scientifically proven medication, and my insomnia has improved significantly, so I don’t feel the need to seek out new forms of treatment like DTx, which is still a relatively new and untested approach.

***Participant I***. *For elderly people who struggle with using digital devices*,* it seems like it would be even more challenging. The idea of being treated with DTx*,* which are based on digital technology rather than medications*,* does not appeal to me. If it weren’t specifically for insomnia DTx*,* I probably would not have been interested at all.*

### Theme 3: Concerns regarding DTx and its platforms

#### Subtheme 3 − 1: Concerns regarding DTx: Cost-effectiveness

The participants expressed that cost-effectiveness was a crucial factor for DTx to be accepted as a viable alternative to conventional interventions. Given that new drugs with updated technologies are generally more expensive, patients are likely to choose oral medication or CBT over DTx, which is already covered by national insurance.

***Participant D***. *It would be great if DTx were more affordable. Since it is not something that you consume*,* like medication or procedures*,* but rather something that works as software*,* the perception of an effective and affordable treatment is important.*

***Participant I***. *When it comes to commercialization*,* financial issues are important. This is partly because psychiatry treatments are generally not covered by insurance as much as other medical fields*,* and also because existing medications are relatively cheaper than DTx. It might be difficult to use DTx if they are more expensive than conventional interventions. Therefore*,* it would be beneficial if DTx could be covered by insurance.*

#### Subtheme 3 − 2: Concerns regarding DTx: Lack of data on treatment efficacy

Several participants expressed doubts regarding the medical efficacy of DTx and felt disappointed if it was simply a digitalized sleep pattern record. Furthermore, they mentioned that it is difficult to comprehend the underlying mechanism of DTx in treating insomnia.

***Participant B*** I am not sure if DTx really has a significant effect. I even have doubts that it may just be a placebo effect. I think it is important to establish its clear and significant effect compared to drug therapy.

***Participant F*** I hope that DTx will be a viable medical treatment option that requires a physician’s prescription, ensuring that its effectiveness is proven. Since it is not just a simple health management product, clinical results are crucial. It is important to demonstrate that it offers significant benefits beyond simply automating sleep-time tracking or digitizing records. All patients should be able to experience meaningful benefits from using DTx.

#### Subtheme 3–3: Concerns regarding platform: Breach of personal information

Most participants expressed concerns about the potential for personal information leaks, especially in terms of consultation content, to other medical institutions and government agencies. However, they were open to sharing objective information, such as a list of prescribed medications or sleep patterns, for reporting of clinical effectiveness and facilitating comfortable use. Moreover, they stated that their medical information could be applied to research as long as their personal data were de-identified.

***Participant B***. *Providing personal information to the attending physician is acceptable*,* but sharing information with other hospitals or insurance companies through DTx can cause discomfort. It would be preferable if medical data could be processed into statistical data that can be used as a reference for treatment.*

***Participant F***. *It would be great to consider some level of convenience when sharing personal information without restricting it too much. In the case of the bank’s personal data project*,* the security was so strict that it made it inconvenient to use.*

***Participant H***. *De-identified personal data can be utilized for research purposes as long as personal identifying information is removed. However*,* there is a concern that the accumulation of data may eventually lead to the re-identification of individuals.*

### Theme 4: Demands regarding DTx and its platforms

#### Subtheme 4 − 1: Demands regarding DTx: convenience and decrease in psychological burden

Most participants acknowledged the positive effects of DTx and its platforms in enhancing convenience during insomnia treatment. These effects included reducing reliance on medication intake, enabling remote monitoring of patient conditions, decreasing the frequency of clinic visits, and providing an alternative treatment option for patients resistant to conventional treatments or those who experience medication-related side effects.

***Participant D*** As a digital platform, it is expected that if the effectiveness is proven, the number of visits to the doctor would decrease and the frequency of medication usage would decrease as well.

***Participant E*** It seems beneficial to divide the treatment into several stages and prescribe it only for mild cases. Additionally, it would be advantageous for patients who are unable to visit the hospital to have the option of a remote prescription or prescription transmission to a pharmacy. Moreover, patients who are averse to medication due to severe side effects would find it favorable.

***Participant G*** In the past, not only did I have to remember to take my medication, but I also had to diligently keep track of my sleep patterns in a personal diary. However, with DTx, it seems that these tasks could be automated, making the treatment process much more convenient and accurate. I believe my primary physician would also have a better understanding of my condition with the help of these digital tools.

#### Subtheme 4 − 2: Demands regarding platforms: compatibility and communication

Most participants expressed their desire for compatibility among different DTx platforms and to enhance communication between patients and physicians. However, they shared negative emotions when small or unlicensed DTx and their associated companies were involved.

***Participant C*** It would be great to be able to use different digital therapies interchangeably if they are compatible with each other. However, I feel like I need to know exactly how many companies are involved in this compatibility. I don’t want to use digital therapies from newly established or strange companies.

***Participant H*** I hope that I can communicate with my healthcare provider through a platform. It would be especially helpful if I could ask questions about the digital therapy I’m using, so that I wouldn’t necessarily have to go to the clinic for simple inquiries and could still receive assistance.

## Discussion

We conducted FGIs with patients with insomnia to examine patients’ recognition, feelings, and demand for the conventional treatments and DTx and its platform. Nine patients with insomnia participated in the FGI to discuss their experiences with conventional insomnia treatments, including difficulties, reasons for inadequate recognition of insomnia DTx, and their concerns and expectations related to DTx and its platform.

Overall, the participants demonstrated limited awareness of DTx and its platforms. Even those who recognized DTx only possessed superficial knowledge, such as awareness of news related to the approval of insomnia DTx in South Korea. The common key point among the participants regarding DTx and DTx platforms was that they could serve as an alternative option for insomnia management. However, little was known about their cost-effectiveness, risk of personal information breach, efficacy, convenience, and compatibility.

Concerns about side effects and dependence on oral medications as well as negative social stigma make it difficult for patients with insomnia to seek conventional psychiatric treatment. Specifically, participants who experienced daytime somnolence, drowsiness, and sleepwalking as side effects of insomnia treatment discontinued the medication. Additionally, given the public’s misunderstanding of conventional psychiatric treatment as a sign of severe mental illness, which was a common difficulty faced by the participants, patients are generally hesitant to seek professional help for insomnia [26]. One participant reported that DTx management is similar to habit management for obesity and that this similarity could help reduce the public’s misunderstanding of psychiatric management. A reduced desire to maintain a distance from mentally ill patients could be helpful in improving help seeking behavior [27].

For these reasons, participants in the study on DTx for insomnia and its platforms identified several features they wanted to see in these platforms, namely enhanced convenience, reduced number of clinic visits, automatic recording of sleep patterns, remote monitoring, low risk of side effects, compatibility among different DTx platforms, communication with medical professionals, and avoidance of social stigma. Because DTx for insomnia is a non-pharmacological and non-invasive treatment, participants expected it to have fewer side effects than traditional treatments, with the exception of the side effects that may be caused by sleep restriction [13]. Overall, DTx and its platforms could be an alternative option for insomnia management due to several advantages, such as convenience, low risk of side effects, and the ability to avoid social stigma.

However, DTx and its platforms must overcome several obstacles. Participants reported that frequent and targeted advertising is needed to raise awareness of the necessity of insomnia management. Additionally, DTx must demonstrate benefits over conventional medical interventions, including medication and CBT, particularly in terms of cost-effectiveness and treatment efficacy. Although the efficacy of digital CBT for insomnia and individual insomnia DTx has been established, building trust in the general population is essential for the widespread adoption of DTx [28, 29]. Specifically, participants expressed doubts about the possibility of DTx being covered by national insurance and the underlying mechanism of DTx.

Furthermore, all participants expressed concerns regarding the breach of personal information during the usage and sharing of medical data due to the involvement of DTx platforms. The Food and Drug Administration (FDA) initiated the regulatory overview for SaMD (a ‘Software Precertification (Pre-Cert) Pilot Program’), designating cybersecurity responsibilities, including potential threats identification and countermeasures development, as primary principle of SaMD [30]. Furthermore, the FDA established guidelines for cybersecurity in medical devices [31]. In South Korea, the cybersecurity level of DTx is classified as a moderate, and it must meet several criteria before release [32]. However, according to one study, only a few DTx platforms have adopted cybersecurity measures [33]. Both DTx and DTx platform developers must communicate their opinions on the cybersecurity criteria to address any concerns.

Meanwhile, one participant residing in a rural area expressed concerns about the scarcity of DTx advertisements. Additionally, the participant also noted challenges faced by older adults who are unfamiliar with digital devices and technology, leading to the lack of access to DTx. This concern is supported by previous studies that have reported the lack of familiarity, information governance, and digital literacy as major barriers to DTx adoption [34]. The participant also expressed doubts regarding the efficacy of DTx but did not state any concerns about social stigma related to psychiatric management. Perhaps, the circumstances in rural areas, including demographic characteristics, availability of digital technology, and subjective perceptions among rural communities toward psychiatric management, are associated with digital/health literacy and DTx availability [35–37]. Further research, considering the geographic location or education level of patients with insomnia, is needed to develop a tailored approach for these patient populations.

## Limitations

This study is the first qualitative investigation on the use of DTx and DTx platforms in patients with insomnia. However, this study has several limitations. First, the homogeneity of the participants was not established, including the age and duration of insomnia symptoms. Second, the interviews were conducted through an online meeting platform, which limited the ability to detect non-verbal cues or atmosphere. Third, the interviews were conducted with insomnia patients in South Korea; therefore, the results cannot be generalized to other countries or diseases. Finally, nine participants were included in the study; therefore, the results may not be representative of all insomnia patients.

## Conclusion

In conclusion, this study explored the subjective difficulties faced by patients with insomnia, the recognition of DTx, obstacles to using DTx, and potential benefits of DTx and DTx platforms. The stakeholders, including healthcare providers, government authorities, and developers, must reflect on and resolve the concerns of patients who use DTx and platforms.

### Electronic supplementary material

Below is the link to the electronic supplementary material.

Supplementary Material 1

Supplementary Material 2

Supplementary Material 3

## Data Availability

No datasets were generated or analysed during the current study.

## Abbreviations

DTX: Digital Therapeutics
SAMD: Software as a Medical Device
MFDS: Ministry of Food And Drug Safety
CBT: Cognitive Behavioral Therapy
FGI: Focus Group Interview
FDA: Food And Drug Administration
