# Supplementary material for: Understanding patient perspectives on digital therapeutics and its platform for insomnia: insights from focused group interviews
Source: BMC Health Serv Res. 2024 Jul 29;24:859. doi: 10.1186/s12913-024-11286-4 (PMC11285125; doi:10.1186/s12913-024-11286-4)
Supplement: Supplementary file 2 — Supplementary Material 2 [file 12913_2024_11286_MOESM2_ESM.docx]

**Article title:** The Understanding of Insomnia Digital Therapeutics and Platform Among Insomnia Patients: A Focused Group Interview Study

**Journal name:** BMC Health Services Research

**Supplementary Table 2** List of the theme, subtheme, and coding tree

| **Theme** | | | **Subtheme** | | |
| --- | --- | --- | --- | --- | --- |
| Experienced subjective difficulty during conventional insomnia treatment | | | Concerns regarding drug side effects and dependence | | |
|  |  |  | Social stigma | | |
|  |  |  | Lack of medical necessity for insomnia management | | |
| The reasons of inadequate recognition status for insomnia DTx | | | Scarcity of advertising of DTx | | |
|  |  |  | Lack of medical necessity for insomnia management | | |
|  |  |  | The presence of conventional medical interventions | | |
| Concerns regarding DTx and DTx platform | | | Concern regarding DTx: Level of cost-effectiveness | | |
|  |  |  | Concern regarding DTx: Unproven treatment efficacy | | |
|  |  |  | Concern regarding platform: Leakage of personal information | | |
| Demands regarding DTx and DTx platform | | | Demand regarding DTx: Convenience and decrease in psychological burden | | |
|  |  |  | Demand regarding platform: Compatibility and Communication | | |
| **Theme** | | **Subtheme** | **Codes** | | **No.** |
| Theme 1 | | Subtheme 1-1 | Oral medication, Side effect, Dependence, Drug resistance | | 4 |
|  |  | Subtheme 1-2 | Mental disorder, Anxiety, Inadequate healthcare policy | | 3 |
|  |  | Subtheme 1-3 | Habitual problem, Transient insomnia, Poor sleep hygiene, Medical symptoms | | 4 |
| Theme 2 | | Subtheme 2-1 | Novel concept, Challenging to comprehend, Rural area | | 3 |
|  |  | Subtheme 2-2 | Perception of severity, Transient insomnia | | 2 |
|  |  | Subtheme 2-3 | Pharmacotherapy, Cognitive behavioral therapy, Demonstrated improvement, Unvalidated treatment option | | 4 |
| Theme 3 | | Subtheme 3-1 | Cost-effectiveness, Expectation of low treatment costs, Health insurance coverage | | 3 |
|  |  | Subtheme 3-2 | Unvalidated therapeutic modality, Unclear underlying mechanism | | 2 |
|  |  | Subtheme 3-3 | Health information, Additional stakeholders involved in information sharing, De-identification | | 3 |
| Theme 4 | | Subtheme 4-1 | Alternative therapeutic modality, Treatment accessibility/convenience, Minimal adverse effects | | 3 |
|  |  | Subtheme 4-2 | Enabled communication, Unregulated/non-approved platform | | 2 |
