# Supplementary material for: Understanding patient perspectives on digital therapeutics and its platform for insomnia: insights from focused group interviews
Source: BMC Health Serv Res. 2024 Jul 29;24:859. doi: 10.1186/s12913-024-11286-4 (PMC11285125; doi:10.1186/s12913-024-11286-4)
Supplement: Supplementary file 3 — Supplementary Material 3 [file 12913_2024_11286_MOESM3_ESM.docx]

**Supplementary Table 3** List of questions and estimated duration during focused group interviews

| **Type of the Question** | **Purpose of the Question** | **Contents** | **Estimated duration** |
| --- | --- | --- | --- |
| **Opening questions** | The purpose of starting with an introductory question that allows all participants to have an opportunity to speak is to create a comfortable atmosphere for discussion and encourage participants to engage in the conversation. | "Please feel free to introduce yourselves, including your name. Additionally, please share with us how long you have been struggling with insomnia" | 2 minutes per each person (Total 10 minutes) |
| **Introduction questions** | The purpose is to encourage participants to consider their relevance to the discussion topic. This involves introducing the topic of discussion and assessing the participants' understanding of the topic. | "Please share with us what you find to be the most challenging aspect of insomnia treatment. For example, this could include difficulties with treatment progress, accessing relevant medical information, understanding explanations from physicians, or reluctance to pursue medication therapy. If you have any personal experiences to share, please feel free to do so." | 3 minutes per each person (Total 15 minutes) |
| **Transition questions** | The role of connecting the introductory and core questions logically is to allow participants to learn how others think about the topic of discussion. This also encourages participants to freely express their thoughts and ideas related to the research topic. | "Recently, a digital therapeutic has received FDA approval in the United States. Are you aware of any digital therapeutics related to insomnia? If so, could you share what you know about them? If not, could you please share your thoughts on what type of service might be available?" | 3 minutes per each person (Total 15 minutes) |
| **Key questions** | As a key component of the research, these questions are central to the analysis and will receive the most focused attention. Therefore, adequate time will be allocated to each question to allow participants to fully discuss their experiences and thoughts. | "What functions do you expect from a digital therapeutic platform, or what concerns do you have? Alternatively, what aspects of the current treatment process do you anticipate would be helped by utilizing such a platform?" | 10 minutes per each person (Total 50 minutes) |
|  |  | (Following detailed questions)  "What do you think about personal information that provided to the digital therapeutic platform and used as public statistical data?" |  |
|  |  | “What do you consider an appropriate level of compatibility and communication among all important participants of DTx platform?" |  |
|  |  | “Why do you think even insomnia patients are unaware of the existence of digital therapeutics, not to mention the digital therapeutics platform?” |  |
|  |  | "Would you be willing to use digital therapeutics and their platforms? If so, what is the reason for your willingness?" |  |
| **Ending questions** | The purpose of this section is to wrap up the discussion and encourage participants to reflect on what they have said. | "Could you please share with us what you think was the most important point from our discussion today?" "Is there anything that you feel we missed or that you would like to add to the discussion?" | 1 minutes per each person (Total 5 minutes) |

Abbreviation: DTx, Digital Therapeutics; FDA, Food and Drug Administration
